# Crystal structure of [2,6-di­fluoro-3-(pyridin-2-yl-κ*N*)pyridin-4-yl-κ*C* ^4^](pentane-2,4-dionato-κ^2^ *O*,*O*′)platinum(II)

**DOI:** 10.1107/S2056989015004375

**Published:** 2015-03-11

**Authors:** Ki-Min Park, Jieun Lee, Youngjin Kang

**Affiliations:** a Research Institute of Natural Science, Gyeongsang National University, Jinju 660-701, Republic of Korea; b Division of Science Education, Kangwon National University, Chuncheon 220-701, Republic of Korea

**Keywords:** crystal structure, platinum(II), CNO_2_ coordination set, hydrogen bonding, π–π inter­actions

## Abstract

The asymmetric unit of the title compound, [Pt(C_10_H_5_F_2_N_2_)(C_5_H_7_O_2_)], comprises one Pt^II^ atom, one 2,6-di­fluoro-2,3-bi­pyridine ligand and one acetyl­acetonate anion. The Pt^II^ atom adopts a distorted square-planar coordination geometry, being *C*,*N*-chelated by the 2,6-di­fluoro-3-(pyridin-2-yl)pyridin-4-yl ligand and *O*,*O*′-chelated by the pentane-2,4-dionate ligand. The two pyridine rings of the bi­pyridine ligand are approximately coplanar, making a dihedral angle of 1.2 (2)°. A variety of intra- and inter­molecular C—H⋯O and C—H⋯F hydrogen bonds, as well as π–π inter­actions [centroid–centroid distances = 4.337 (3) and 3.774 (3) Å] contribute to the stabilization of the mol­ecular and crystal structures, and result in the formation of a three-dimensional supra­molecular framework.

## Abstract 2

The Pt^II^ atom adopts a distorted square-planar coordination geometry, being *C*,*N*-chelated by a 2′,6′-di­fluoro-2,3′-bi­pyridine ligand and *O*,*O′*-chelated by a pentane-2,4-dionato anionic ligand.

## Chemical context   

Cyclo­metalated platinum(II) compounds with *C,N*-chelating ligands have been considered as an attractive research area due to their wide applications, such as biological imaging, non-linear optics, oxygen sensing and organic light-emitting diodes (OLEDs) (Hudson *et al.*, 2012 ▸). In particular, phenyl­pyridine (ppy) based platinum(II) *β*-diketonate compounds have been widely studied because of their excellent stability and high quantum efficiency in OLEDs (Rao *et al.*, 2012 ▸). However, examples of platinum(II) compounds with *C*,*N*-chelating bi­pyridine ligands are scarce. Herein, we report the result of our investigation on the crystal structure of a novel platinum(II) compound with fluorinated bi­pyridine and acetyl­acetonate (acac, *O*,*O*) ligands.
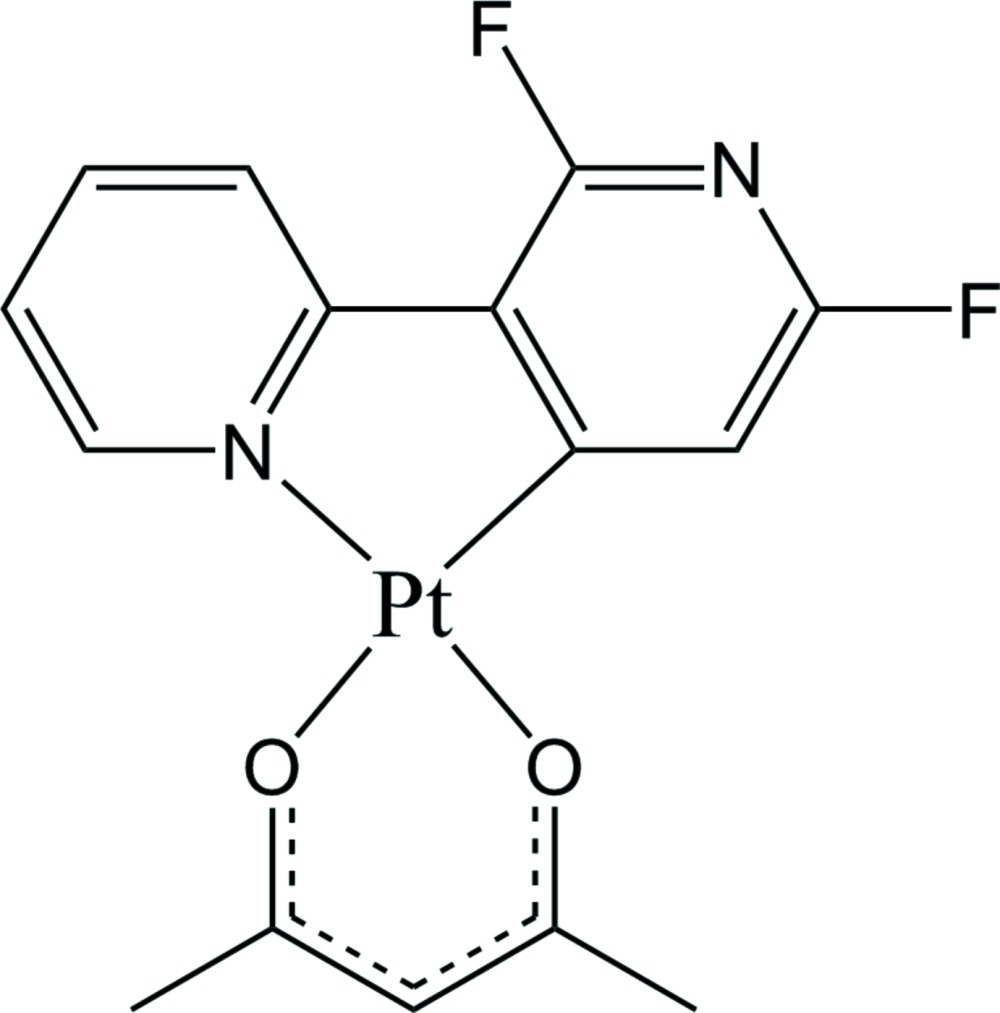


## Structural commentary   

**Figure 1:**
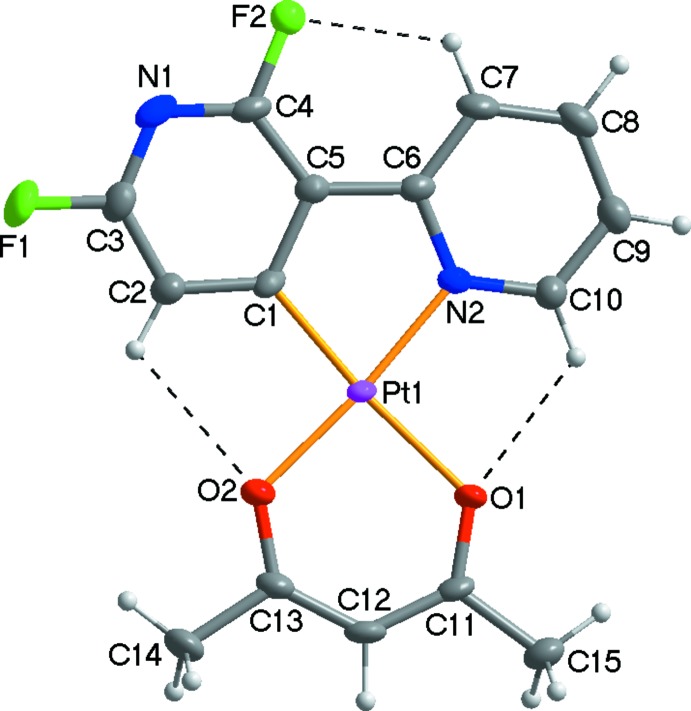
View of the mol­ecular structure of the title compound, with the atom-numbering scheme. Displacement ellipsoids are drawn at the 50% probability level; dashed lines represent intra­molecular C—H⋯O and C—H⋯F hydrogen bonds.

The mol­ecular structure of the title compound is shown in Fig. 1 ▸. The asymmetric unit consists of one Pt^II^ atom, one 2,6-di­fluoro-2,3-bi­pyridine ligand and one acetyl­acetonate anion. The Pt^II^ atom is four-coordinated by the *C*,*N*-chelating 2′,6′-di­fluoro-2,3′-bipyridinato ligand and by the *O*,*O′*-chelating pentane-2,4-dionato ligand, forming a distorted square-planar coordination sphere due to narrow ligand bite angles, which range from 81.28 (17) to 93.25 (13)°. The Pt—C bond length of 1.951 (4) Å is shorter than the Pt—N bond length of 1.995 (4) Å due to the more electronegative fluorine substit­uent on the C-bound pyridine ring. The Pt—C, Pt—N and Pt—O bond lengths (Table 1 ▸) are in normal ranges as reported for similar Pt^II^ compounds, *e.g*. [Pt(Bppy)(acac)] (Bppy is a boron-functionalized phenyl­pyridine; Rao *et al.*, 2012 ▸). Within the *C*,*N*-bidentate ligand of the title compound, the two pyridine rings are approximately co-planar, making a dihedral angle of 1.2 (2)°, indicating that an effective π conjugation of the two pyridine rings occurs in the title compound. The mol­ecular structure is stabilized by weak intra­molecular C—H⋯O and C—H⋯F hydrogen bonds (Table 2 ▸).

## Supra­molecular features   

**Figure 2:**
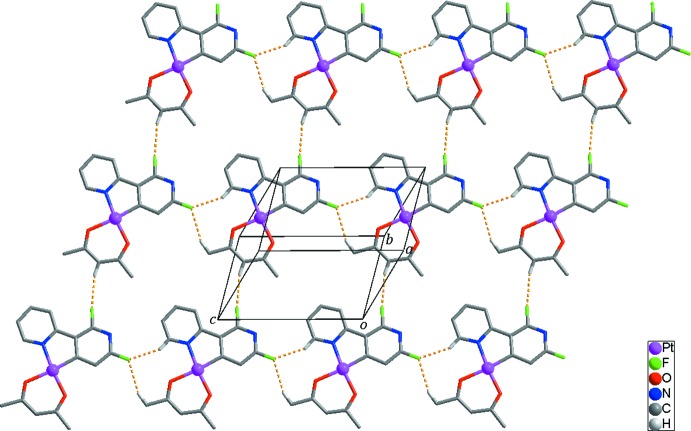
The two-dimensional supra­molecular network formed through C—H⋯F inter­actions (yellow dashed lines). H atoms not involved in inter­molecular inter­actions have been omitted for clarity.

**Figure 3:**
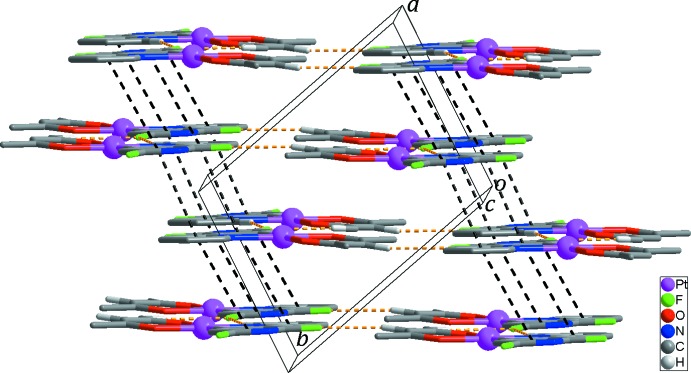
The three-dimensional supra­molecular network formed through π–π stacking inter­actions (black dashed lines). Yellow dashed lines indicate the C—H⋯F inter­actions. H atoms not involved in inter­molecular inter­actions have been omitted for clarity.

Inter­molecular C—H⋯F hydrogen bonds between neighboring mol­ecules lead to the formation of a two-dimensional supra­molecular network extending parallel to the (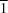
10) plane (Fig. 2 ▸, Table 2 ▸). These networks are inter­linked by π–π inter­actions [*Cg*1—*Cg*2^i^ = 4.337 (3) Å and *Cg*1—*Cg*2^ii^ = 3.774 (3) Å, where *Cg*1 and *Cg*2 are the centroids of the N1, C1–C5 and the N2, C6–C10 rings, respectively; symmetry codes: (i) −*x* + 1, −*y* + 2, −*z* + 2; (ii) −*x* + 2, −*y* + 2, −*z* + 2], resulting in the formation of an overall three-dimensional supra­molecular framework (Fig. 3 ▸).

## Synthesis and crystallization   

The title compound was synthesized according to a previous report (Rao *et al.*, 2012 ▸). Slow evaporation from a di­chloro­methane/hexane solution afforded yellow crystals suitable for X-ray crystallography analysis.

## Refinement   

Crystal data, data collection and crystal structure refinement details are summarized in Table 3 ▸. All H atoms were positioned geometrically and refined using a riding model, with *d*(C—H) = 0.95 Å, *U*_iso_(H) = 1.2*U*_eq_(C) for C*sp* ^2^-H, and 0.98 Å, *U*_iso_(H) = 1.5*U*_eq_(C) for methyl protons.

## Supplementary Material

Crystal structure: contains datablock(s) I, New_Global_Publ_Block. DOI: 10.1107/S2056989015004375/wm5128sup1.cif

Structure factors: contains datablock(s) I. DOI: 10.1107/S2056989015004375/wm5128Isup2.hkl

CCDC reference: 1051825

Additional supporting information:  crystallographic information; 3D view; checkCIF report

## Figures and Tables

**Table 1:** Selected bond lengths ()

Pt1C1	1.951(4)	Pt1O1	2.074(3)
Pt1N2	1.995(4)	Pt1O2	2.001(3)

**Table 2:** Hydrogen-bond geometry (, )

*D*H*A*	*D*H	H*A*	*D* *A*	*D*H*A*
C2H2O2	0.95	2.57	3.040(5)	111
C7H7F2	0.95	2.31	2.917(6)	121
C10H10F1^i^	0.95	2.32	3.180(5)	150
C10H10O1	0.95	2.41	3.006(5)	120
C12H12F2^ii^	0.95	2.44	3.361(5)	163
C15H15*A*F1^i^	0.98	2.54	3.481(6)	161

Symmetry codes: (i) 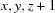; (ii) 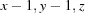.

**Table 3:** Experimental details

Crystal data	
Chemical formula	[Pt(C_10_H_5_F_2_N_2_)(C_5_H_7_O_2_)]
*M*_r_	485.36
Crystal system, space group	Triclinic, *P* 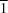
Temperature (K)	180
*a*, *b*, *c* ()	8.0442(6), 9.8711(7), 10.1458(7)
, , ()	97.683(1), 112.320(1), 99.410(1)
*V* (^3^)	718.12(9)
*Z*	2
Radiation type	Mo *K*
(mm^1^)	9.80
Crystal size (mm)	0.27 0.24 0.12

Data collection	
Diffractometer	Bruker APEXII CCD area detector
Absorption correction	Multi-scan (*SADABS*; Bruker, 2006 ▸)
*T*_min_, *T*_max_	0.177, 0.386
No. of measured, independent and observed [*I* > 2(*I*)] reflections	7062, 2810, 2773
*R*_int_	0.023
(sin /)_max_ (^1^)	0.617

Refinement	
*R*[*F* ^2^ > 2(*F* ^2^)], *wR*(*F* ^2^), *S*	0.017, 0.053, 1.07
No. of reflections	2810
No. of parameters	199
H-atom treatment	H-atom parameters constrained
_max_, _min_ (e ^3^)	0.51, 1.27

Computer programs: *APEX2* and *SAINT* (Bruker, 2006 ▸), *SHELXS97*, *SHELXL97* and *SHELXTL* (Sheldrick, 2008 ▸) and *DIAMOND* (Brandenburg, 2005 ▸).

## supplementary crystallographic information

### Crystal data

**Table:**

[Pt(C_10_H_5_F_2_N_2_)(C_5_H_7_O_2_)]	*Z* = 2
*M**_r_* = 485.36	*F*(000) = 456
Triclinic, *P*1	*D*_x_ = 2.245 Mg m^−^^3^
Hall symbol: -P 1	Mo *K*α radiation, λ = 0.71073 Å
*a* = 8.0442 (6) Å	Cell parameters from 2773 reflections
*b* = 9.8711 (7) Å	θ = 2.1–26.0°
*c* = 10.1458 (7) Å	µ = 9.80 mm^−^^1^
α = 97.683 (1)°	*T* = 180 K
β = 112.320 (1)°	Block, yellow
γ = 99.410 (1)°	0.27 × 0.24 × 0.12 mm
*V* = 718.12 (9) Å^3^	

### Data collection

**Table:**

Bruker APEXII CCD area-detector diffractometer	2810 independent reflections
Radiation source: fine-focus sealed tube	2773 reflections with *I* > 2σ(*I*)
Graphite monochromator	*R*_int_ = 0.023
φ and ω scans	θ_max_ = 26.0°, θ_min_ = 2.1°
Absorption correction: multi-scan (*SADABS*; Bruker, 2006)	*h* = −9→9
*T*_min_ = 0.177, *T*_max_ = 0.386	*k* = −12→12
7062 measured reflections	*l* = −12→12

### Refinement

**Table:**

Refinement on *F*^2^	Primary atom site location: structure-invariant direct methods
Least-squares matrix: full	Secondary atom site location: difference Fourier map
*R*[*F*^2^ > 2σ(*F*^2^)] = 0.017	Hydrogen site location: inferred from neighbouring sites
*wR*(*F*^2^) = 0.053	H-atom parameters constrained
*S* = 1.07	*w* = 1/[σ^2^(*F*_o_^2^) + (0.0295*P*)^2^ + 1.5655*P*] where *P* = (*F*_o_^2^ + 2*F*_c_^2^)/3
2810 reflections	(Δ/σ)_max_ = 0.002
199 parameters	Δρ_max_ = 0.51 e Å^−^^3^
0 restraints	Δρ_min_ = −1.27 e Å^−^^3^

### Special details

**Table:**

Geometry. All e.s.d.'s (except the e.s.d. in the dihedral angle between two l.s. planes) are estimated using the full covariance matrix. The cell e.s.d.'s are taken into account individually in the estimation of e.s.d.'s in distances, angles and torsion angles; correlations between e.s.d.'s in cell parameters are only used when they are defined by crystal symmetry. An approximate (isotropic) treatment of cell e.s.d.'s is used for estimating e.s.d.'s involving l.s. planes.
Refinement. Refinement of *F*^2^ against ALL reflections. The weighted *R*-factor *wR* and goodness of fit *S* are based on *F*^2^, conventional *R*-factors *R* are based on *F*, with *F* set to zero for negative *F*^2^. The threshold expression of *F*^2^ > σ(*F*^2^) is used only for calculating *R*-factors(gt) *etc*. and is not relevant to the choice of reflections for refinement. *R*-factors based on *F*^2^ are statistically about twice as large as those based on *F*, and *R*- factors based on ALL data will be even larger.

### Fractional atomic coordinates and isotropic or equivalent isotropic displacement parameters (Å^2^)

**Table:**

	*x*	*y*	*z*	*U*_iso_*/*U*_eq_	
Pt1	0.541428 (18)	0.778516 (13)	0.974446 (14)	0.01645 (7)	
F1	0.6043 (5)	0.8519 (4)	0.4799 (3)	0.0462 (8)	
F2	0.9252 (4)	1.1982 (3)	0.8748 (3)	0.0362 (6)	
O1	0.4622 (4)	0.7037 (3)	1.1284 (3)	0.0240 (6)	
O2	0.3730 (4)	0.6133 (3)	0.8158 (3)	0.0231 (6)	
N1	0.7621 (5)	1.0252 (4)	0.6783 (4)	0.0294 (8)	
N2	0.7143 (5)	0.9517 (4)	1.1169 (4)	0.0197 (7)	
C1	0.6273 (6)	0.8664 (4)	0.8434 (5)	0.0209 (8)	
C2	0.5731 (6)	0.8150 (5)	0.6931 (5)	0.0251 (8)	
H2	0.4892	0.7267	0.6431	0.030*	
C3	0.6477 (6)	0.8990 (5)	0.6230 (5)	0.0290 (9)	
C4	0.8076 (6)	1.0704 (4)	0.8184 (5)	0.0245 (8)	
C5	0.7490 (5)	0.9996 (4)	0.9079 (4)	0.0205 (8)	
C6	0.7995 (6)	1.0466 (4)	1.0634 (5)	0.0210 (8)	
C7	0.9196 (6)	1.1711 (4)	1.1559 (5)	0.0263 (9)	
H7	0.9801	1.2372	1.1190	0.032*	
C8	0.9507 (6)	1.1986 (5)	1.3023 (5)	0.0295 (9)	
H8	1.0316	1.2836	1.3659	0.035*	
C9	0.8626 (6)	1.1006 (5)	1.3545 (5)	0.0279 (9)	
H9	0.8829	1.1168	1.4544	0.034*	
C10	0.7447 (6)	0.9790 (5)	1.2585 (5)	0.0249 (8)	
H10	0.6829	0.9121	1.2937	0.030*	
C11	0.3374 (6)	0.5912 (4)	1.0981 (5)	0.0219 (8)	
C12	0.2383 (6)	0.5027 (4)	0.9600 (5)	0.0247 (9)	
H12	0.1450	0.4256	0.9525	0.030*	
C13	0.2611 (6)	0.5149 (4)	0.8326 (5)	0.0230 (8)	
C14	0.1488 (7)	0.4045 (5)	0.6953 (5)	0.0328 (10)	
H14A	0.1816	0.4288	0.6163	0.049*	
H14B	0.0170	0.3996	0.6684	0.049*	
H14C	0.1746	0.3131	0.7117	0.049*	
C15	0.3027 (7)	0.5529 (5)	1.2257 (5)	0.0305 (10)	
H15A	0.3822	0.6245	1.3142	0.046*	
H15B	0.3306	0.4613	1.2389	0.046*	
H15C	0.1728	0.5479	1.2072	0.046*	

### Atomic displacement parameters (Å^2^)

**Table:**

	*U*^11^	*U*^22^	*U*^33^	*U*^12^	*U*^13^	*U*^23^
Pt1	0.01828 (10)	0.01327 (9)	0.01850 (9)	0.00005 (6)	0.00925 (7)	0.00501 (6)
F1	0.062 (2)	0.0564 (19)	0.0253 (14)	0.0043 (16)	0.0257 (14)	0.0124 (13)
F2	0.0389 (15)	0.0238 (13)	0.0495 (17)	−0.0032 (11)	0.0244 (13)	0.0138 (12)
O1	0.0251 (15)	0.0198 (14)	0.0209 (14)	−0.0073 (12)	0.0087 (12)	0.0017 (11)
O2	0.0242 (14)	0.0179 (14)	0.0241 (14)	−0.0014 (11)	0.0095 (12)	0.0034 (11)
N1	0.031 (2)	0.033 (2)	0.034 (2)	0.0088 (16)	0.0200 (17)	0.0187 (17)
N2	0.0176 (16)	0.0169 (16)	0.0235 (17)	0.0015 (13)	0.0083 (14)	0.0045 (13)
C1	0.029 (2)	0.0176 (19)	0.025 (2)	0.0086 (16)	0.0169 (18)	0.0103 (16)
C2	0.027 (2)	0.025 (2)	0.022 (2)	0.0021 (17)	0.0101 (17)	0.0073 (16)
C3	0.032 (2)	0.037 (2)	0.021 (2)	0.0097 (19)	0.0124 (18)	0.0108 (18)
C4	0.023 (2)	0.021 (2)	0.035 (2)	0.0044 (16)	0.0161 (18)	0.0130 (17)
C5	0.0191 (18)	0.0190 (19)	0.025 (2)	0.0043 (15)	0.0102 (16)	0.0065 (16)
C6	0.0198 (18)	0.0182 (19)	0.028 (2)	0.0041 (15)	0.0119 (17)	0.0077 (16)
C7	0.023 (2)	0.0170 (19)	0.037 (2)	0.0004 (16)	0.0115 (18)	0.0070 (17)
C8	0.025 (2)	0.021 (2)	0.031 (2)	−0.0002 (17)	0.0042 (18)	−0.0021 (17)
C9	0.029 (2)	0.027 (2)	0.022 (2)	0.0038 (18)	0.0074 (17)	−0.0002 (17)
C10	0.028 (2)	0.024 (2)	0.023 (2)	0.0032 (17)	0.0120 (17)	0.0037 (16)
C11	0.023 (2)	0.020 (2)	0.028 (2)	0.0037 (16)	0.0136 (17)	0.0129 (16)
C12	0.021 (2)	0.0175 (19)	0.034 (2)	−0.0024 (16)	0.0119 (18)	0.0086 (17)
C13	0.0207 (19)	0.0160 (19)	0.027 (2)	−0.0004 (15)	0.0065 (17)	0.0040 (16)
C14	0.031 (2)	0.025 (2)	0.029 (2)	−0.0074 (18)	0.0057 (19)	−0.0023 (18)
C15	0.030 (2)	0.032 (2)	0.032 (2)	0.0008 (19)	0.016 (2)	0.0124 (19)

### Geometric parameters (Å, º)

**Table:**

Pt1—C1	1.951 (4)	C7—C8	1.389 (7)
Pt1—N2	1.995 (4)	C7—H7	0.9500
Pt1—O1	2.074 (3)	C8—C9	1.384 (7)
Pt1—O2	2.001 (3)	C8—H8	0.9500
F1—C3	1.352 (5)	C9—C10	1.379 (6)
F2—C4	1.349 (5)	C9—H9	0.9500
O1—C11	1.282 (5)	C10—H10	0.9500
O2—C13	1.288 (5)	C11—C12	1.399 (6)
N1—C4	1.316 (6)	C11—C15	1.506 (6)
N1—C3	1.327 (6)	C12—C13	1.391 (6)
N2—C10	1.343 (5)	C12—H12	0.9500
N2—C6	1.361 (5)	C13—C14	1.504 (6)
C1—C5	1.406 (6)	C14—H14A	0.9800
C1—C2	1.410 (6)	C14—H14B	0.9800
C2—C3	1.368 (6)	C14—H14C	0.9800
C2—H2	0.9500	C15—H15A	0.9800
C4—C5	1.387 (6)	C15—H15B	0.9800
C5—C6	1.457 (6)	C15—H15C	0.9800
C6—C7	1.393 (6)		
			
C1—Pt1—N2	81.28 (17)	C6—C7—H7	120.0
C1—Pt1—O2	92.90 (16)	C9—C8—C7	119.3 (4)
N2—Pt1—O2	174.15 (12)	C9—C8—H8	120.4
C1—Pt1—O1	174.40 (14)	C7—C8—H8	120.4
N2—Pt1—O1	93.25 (13)	C10—C9—C8	118.6 (4)
O2—Pt1—O1	92.55 (12)	C10—C9—H9	120.7
C11—O1—Pt1	123.4 (3)	C8—C9—H9	120.7
C13—O2—Pt1	123.9 (3)	N2—C10—C9	122.4 (4)
C4—N1—C3	113.8 (4)	N2—C10—H10	118.8
C10—N2—C6	119.9 (4)	C9—C10—H10	118.8
C10—N2—Pt1	123.4 (3)	O1—C11—C12	125.5 (4)
C6—N2—Pt1	116.7 (3)	O1—C11—C15	115.4 (4)
C5—C1—C2	117.9 (4)	C12—C11—C15	119.1 (4)
C5—C1—Pt1	114.5 (3)	C13—C12—C11	127.3 (4)
C2—C1—Pt1	127.4 (3)	C13—C12—H12	116.4
C3—C2—C1	116.6 (4)	C11—C12—H12	116.4
C3—C2—H2	121.7	O2—C13—C12	127.1 (4)
C1—C2—H2	121.7	O2—C13—C14	113.0 (4)
N1—C3—F1	113.5 (4)	C12—C13—C14	119.9 (4)
N1—C3—C2	127.9 (4)	C13—C14—H14A	109.5
F1—C3—C2	118.6 (4)	C13—C14—H14B	109.5
N1—C4—F2	113.7 (4)	H14A—C14—H14B	109.5
N1—C4—C5	126.6 (4)	C13—C14—H14C	109.5
F2—C4—C5	119.7 (4)	H14A—C14—H14C	109.5
C4—C5—C1	117.2 (4)	H14B—C14—H14C	109.5
C4—C5—C6	127.6 (4)	C11—C15—H15A	109.5
C1—C5—C6	115.3 (4)	C11—C15—H15B	109.5
N2—C6—C7	119.9 (4)	H15A—C15—H15B	109.5
N2—C6—C5	112.2 (4)	C11—C15—H15C	109.5
C7—C6—C5	128.0 (4)	H15A—C15—H15C	109.5
C8—C7—C6	119.9 (4)	H15B—C15—H15C	109.5
C8—C7—H7	120.0		
			
N2—Pt1—O1—C11	176.1 (3)	C2—C1—C5—C6	180.0 (4)
O2—Pt1—O1—C11	−3.1 (3)	Pt1—C1—C5—C6	3.4 (5)
C1—Pt1—O2—C13	−175.2 (3)	C10—N2—C6—C7	0.7 (6)
O1—Pt1—O2—C13	3.5 (3)	Pt1—N2—C6—C7	178.9 (3)
C1—Pt1—N2—C10	−179.3 (4)	C10—N2—C6—C5	−179.6 (4)
O1—Pt1—N2—C10	1.9 (3)	Pt1—N2—C6—C5	−1.4 (4)
C1—Pt1—N2—C6	2.6 (3)	C4—C5—C6—N2	179.1 (4)
O1—Pt1—N2—C6	−176.2 (3)	C1—C5—C6—N2	−1.3 (5)
N2—Pt1—C1—C5	−3.2 (3)	C4—C5—C6—C7	−1.2 (7)
O2—Pt1—C1—C5	176.2 (3)	C1—C5—C6—C7	178.4 (4)
N2—Pt1—C1—C2	−179.4 (4)	N2—C6—C7—C8	−0.6 (6)
O2—Pt1—C1—C2	0.0 (4)	C5—C6—C7—C8	179.8 (4)
C5—C1—C2—C3	1.4 (6)	C6—C7—C8—C9	0.5 (6)
Pt1—C1—C2—C3	177.4 (3)	C7—C8—C9—C10	−0.6 (7)
C4—N1—C3—F1	−178.8 (4)	C6—N2—C10—C9	−0.8 (6)
C4—N1—C3—C2	0.9 (7)	Pt1—N2—C10—C9	−178.9 (3)
C1—C2—C3—N1	−1.8 (7)	C8—C9—C10—N2	0.8 (7)
C1—C2—C3—F1	177.9 (4)	Pt1—O1—C11—C12	0.5 (6)
C3—N1—C4—F2	179.4 (4)	Pt1—O1—C11—C15	178.7 (3)
C3—N1—C4—C5	0.3 (6)	O1—C11—C12—C13	3.4 (7)
N1—C4—C5—C1	−0.6 (6)	C15—C11—C12—C13	−174.8 (4)
F2—C4—C5—C1	−179.6 (4)	Pt1—O2—C13—C12	−1.3 (6)
N1—C4—C5—C6	179.1 (4)	Pt1—O2—C13—C14	179.2 (3)
F2—C4—C5—C6	0.0 (6)	C11—C12—C13—O2	−3.0 (7)
C2—C1—C5—C4	−0.3 (6)	C11—C12—C13—C14	176.4 (4)
Pt1—C1—C5—C4	−176.9 (3)		

### Hydrogen-bond geometry (Å, º)

**Table:**

*D*—H···*A*	*D*—H	H···*A*	*D*···*A*	*D*—H···*A*
C2—H2···O2	0.95	2.57	3.040 (5)	111
C7—H7···F2	0.95	2.31	2.917 (6)	121
C10—H10···F1^i^	0.95	2.32	3.180 (5)	150
C10—H10···O1	0.95	2.41	3.006 (5)	120
C12—H12···F2^ii^	0.95	2.44	3.361 (5)	163
C15—H15*A*···F1^i^	0.98	2.54	3.481 (6)	161

Symmetry codes: (i) *x*, *y*, *z*+1; (ii) *x*−1, *y*−1, *z*.
